# Why animal model studies are lost in translation

**DOI:** 10.20517/jca.2022.10

**Published:** 2022-03-31

**Authors:** Nikolaos G. Frangogiannis

**Affiliations:** The Wilf Family Cardiovascular Research Institute, Department of Medicine (Cardiology), Albert Einstein College of Medicine, Bronx, NY 10461, USA.

**Keywords:** Animal model, translation, pathophysiology, human disease

## Abstract

The development of novel therapies based on understanding the pathophysiologic basis of disease is a major goal of biomedical research. Despite an explosion in new knowledge on the molecular mechanisms of disease derived from animal model investigations, translation into effective treatment for human patients has been disappointingly slow. Several fundamental problems may explain the translational failures. First, the emphasis on novel and highly significant findings selectively rewards implausible, low-probability observations and high-magnitude effects, providing a biased perspective of the pathophysiology of disease that underappreciates the complexity and redundancy of biological systems. Second, even when a sound targetable mechanism is identified, animal models cannot recapitulate the pathophysiologic heterogeneity of the human disease, and are poor predictors of therapeutic success. Third, traditional classifications of most complex diseases are based primarily on clinical criteria and do not reflect the diverse pathophysiologic mechanisms that may be involved. The development of a flexible and dynamic conceptual paradigm that takes into account the totality of the evidence on the mechanisms of disease, and pathophysiologic stratification of patients to identify subpopulations with distinct pathogenetic mechanisms, are crucial for the development of new therapeutics.

The development of successful therapeutic approaches requires insights into the mechanisms of disease. Historically, the effectiveness of medical therapeutics has been dependent on the level of understanding of the pathophysiologic basis of disease. In the neolithic era, procedures drilling holes into the skull (known as trepanning) were used as therapy for a wide range of conditions, based on the premise that malicious spirits were the main cause of every disease. The Hellenic civilization revolutionized the conceptual basis of medicine, by suggesting that all illnesses have natural causes that do not involve metaphysical or supernatural interventions. Despite the brilliant philosophical concepts introduced by the ancient Greeks, their primitive technology and underdeveloped experimental approaches greatly limited their ability to gain pathophysiologic insights and advance therapeutics. In the modern era, the explosive growth of knowledge in biology, biochemistry and biophysics and the extensive use of animal models provide a wide range of new tools to develop novel therapeutics based on pathophysiologic insights. Over the last 30 years, seemingly robust experimental studies have revealed a myriad of critical molecular pathways mediating the most common diseases, from cancer to atherosclerotic disease, and from heart failure to chronic neurological disorders. In many cases, interventions targeting these pathways in animal disease models have suggested impressive beneficial effects. Unfortunately, effective approaches in animal models are seldom followed by therapeutic success in human patients.

What is the reason for these translational failures? This viewpoint manuscript discusses the basis for the disconnect between the amazing successes of animal model research and the paucity of effective strategies in human patients, and emphasizes the need for a re-appraisal of our interpretation of research findings. The expectations for highly effective new therapies are often based on a biased perspective, driven by publication and funding priorities that tend to reward implausible, low-probability experimental findings, thus painting an oversimplified and distorted view of the pathophysiology of disease. Instead of relying on single “high-impact” studies to identify therapeutic targets, a dynamic conceptual paradigm informed by the totality of the evidence in the field should drive translational efforts. Second, considering their pathophysiologic heterogeneity, most complex human diseases cannot be recapitulated by a single animal model. In most cases, animal models should be viewed primarily as tools for pathophysiologic dissection of a cell biological response relevant to the human disease, rather than as direct predictors of clinical outcome in human patients. Third, traditional clinical definitions of common multifactorial illnesses are not helpful for the design of pathophysiologically-driven interventions. There is a need for pathophysiologic stratification of common diseases, in order to identify patient subpopulations with common molecular defects or cell biological perturbations.

## Painting the landscape: the use of animal models to understand the pathophysiologic basis of disease

Identification of molecular mechanisms with a causative role in the pathogenesis of disease requires experimental interventions in well-characterized experimental models that recapitulate relevant cell biological aspects of the pathophysiology of the disease. Genetic loss-and gain-of-function strategies^[1,2]^ have revolutionized pathophysiologic dissection, allowing conditional cell-specific ablation, or overexpression of proteins encoded by specific genes, in order to understand their role in the cellular response of interest, and their contribution to the functional abnormality. Experimental studies have identified a long list of molecular signals with critical roles in the pathogenesis of various diseases. Based on these studies, a broad range of therapeutic targets have been identified. Unfortunately, despite a proliferation in published findings, understanding of the pathophysiology of most common diseases remains limited, and translation of specific findings into effective therapeutic strategies for human patients has been disappointingly slow. Many highly promising strategies with impressive beneficial effects in animal models have failed to show effectiveness in the clinic^[3,4]^. Considering the high cost and extensive resources required to test and develop a new therapeutic strategy, the large number of seemingly promising directions poses a major challenge for clinical translation. Do experimental studies provide us with a useful roadmap towards translation?

## The well-justified emphasis on innovation and significance of the findings produces a bias favoring the publication of improbable positive observations

**Figure 1.**
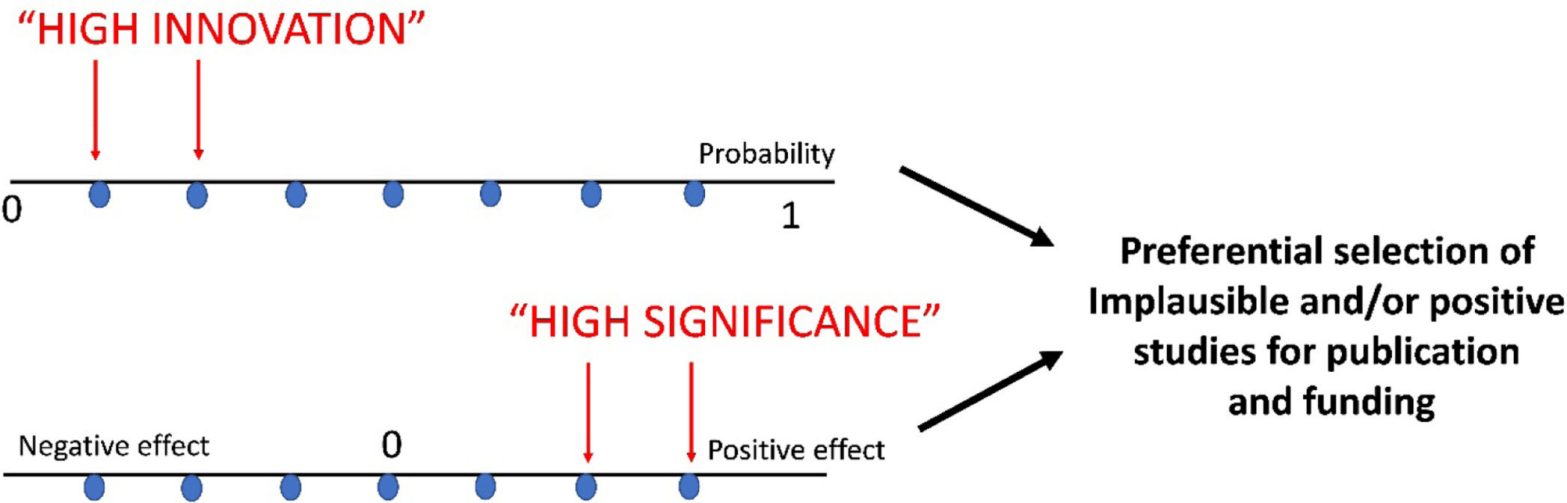
The (well-justified) emphasis on innovation and significance tends to favor publication and funding of improbable observations with impressive positive results. Typically, surprising findings that challenge existing concepts and support a mechanism with a low pre-study probability, are perceived as novel, and are more likely to be published in high-impact journals. Moreover, interventional studies are considered “highly significant” when strongly positive effects are found. Studies perceived as highly innovative and/or highly significant (red arrows) are selectively published, and are also more likely to attract funding. Moreover, these competitive advantages of high innovation/high-significance findings exert pressures on success-driven investigators that may generate additional investigator-dependent intentional or non-intentional bias. In contrast, findings supporting more plausible, high-probability concepts, or interventions producing modest or negative effects are considered much less exciting, have a lower chance of publication in high-impact journals, and may not attract research funding. These patterns in publication priority, research funding and dissemination of study results paint a biased perspective of a field, disproportionately rewarding improbable observations that report high-magnitude effects.

High innovation and outstanding significance are the overarching criteria for high-impact publications. Although the high publication priority of novel and important findings is obviously well-justified, the quest of the scientific community for newsworthy stories has some unwanted consequences, especially when it is not accompanied by an emphasis on rigorous data [Figure 1]. Observations perceived as “novel” typically describe unusual and surprising low-probability events that may challenge existing concepts. These improbable observations have a higher chance of publication in a high-impact journal than findings that confirm the existing paradigm. Moreover, investigators reporting improbable observations are more likely to attract funding in order to pursue their research. Thus, the high rewards of low probability findings (based on their perceived novelty) create a bias favoring their publication and dissemination.

Moreover, the emphasis on highly significant observations introduces an additional bias towards findings that suggest strongly positive effects, rather than neutral studies. Ultimately, these selection advantages result in preferential publication and dissemination of positive studies reporting impressive results that have a lower probability of being correct. In contrast, experimental studies that simply confirm an established concept, or negative studies that fail to reveal a significant effect of a mediator or a molecular pathway attract much less attention, and fail to generate funding. Such studies often remain incomplete, or are typically published in lower impact journals.

The final result of this pattern of scientific sensationalism is that the scientific community has become almost immune to reports suggesting impressive effects of various interventions in animal models. Cure of challenging and complex multifactorial diseases (such as atherosclerosis, obesity and diabetes, and heart failure) in mouse models has become commonplace. For example, in the field of myocardial ischemic injury, there is a very long list of genetic and pharmacologic interventions that have been reported to markedly reduce infarct size in animal models of myocardial ischemia. Although the discovery of only one truly effective infarct size-reducing approach would have been expected to generate great enthusiasm in the field, due to its potentially transformative impact on the treatment of patients with myocardial infarction, new findings reporting impressive protective effects in ischemia models are nowadays treated almost as routine observations. This is due to the untold, but highly prevalent conviction of many members of the scientific community that the vast majority of these findings are probably not true^[5]^, or at least cannot be generalized beyond the very specific conditions studied by the authors. Clearly, this alarming reality poses major challenges for effective translation, as identification of truly effective therapeutic targets within a myriad of highly promising (but probably overrated) approaches becomes impossible.

## The translation should be driven by a dynamic and flexible cell biological paradigm taking into account the totality of the evidence and not by single studies

Considering the pathophysiologic complexity of most common diseases, the translation should be pursued through the development of a general cell biological paradigm that attempts to explain the pathologic response by incorporating the totality of the evidence, and not through the adoption of specific concepts suggested by single investigations. A well-developed pathophysiologic paradigm operates under a set of assumptions on the events governing cellular behavior, tissue structure and organ function. These assumptions are based on evidence from several different sources: animal models, cell biological experiments and descriptive human studies. A pathophysiologic paradigm is highly dynamic and flexible, capable of adapting to include new findings and discoveries. The paradigm should also adapt to changes in the course and characteristics of the disease that may be driven by changes in the environment or alterations in the prevalence of predisposing conditions. Every new study is interpreted within the spectrum of cellular and molecular mechanisms suggested by the existing paradigm. Paradigmatic shifts can occur, but are rare. No single study can decisively shift the model; however, the cumulative weight of all published evidence shapes the prevailing concepts.

## Animal models should be predominantly used for dissection of pathophysiologic mechanisms, and not as predictors of therapeutic effectiveness

A typical approach in the quest towards translation is to directly examine whether the new therapeutic strategy improves clinical outcome, or ameliorates dysfunction in an animal model of the disease. However, in most complex human pathologic conditions, this approach is highly problem0061tic. Several major limitations reduce the value of animal models in providing direct insights into the therapeutic effectiveness of an approach, even when studying interventions on molecular targets without significant species specificity in their biological effects. First, because a broad spectrum of perturbations can result in the same clinical syndrome, no animal model can recapitulate the range of different pathologies observed in a human disease. An animal model is most valuable when it recapitulates a specific cell biological response of high relevance in the human disease of interest.

Second, measures of clinical outcome do not necessarily translate well from animal models to human patients. Mortality in the human disease may be caused by different cellular mechanisms than in the animal model, making interpretation of outcome data particularly challenging. For example, in non-reperfused myocardial infarction, mice exhibit high early mortality due to cardiac rupture, an uncommon cause of death in human patients. In contrast, infarcted mice seem to tolerate low output states much better than humans, and may exhibit a lower incidence of arrhythmias.

**Figure 2.**
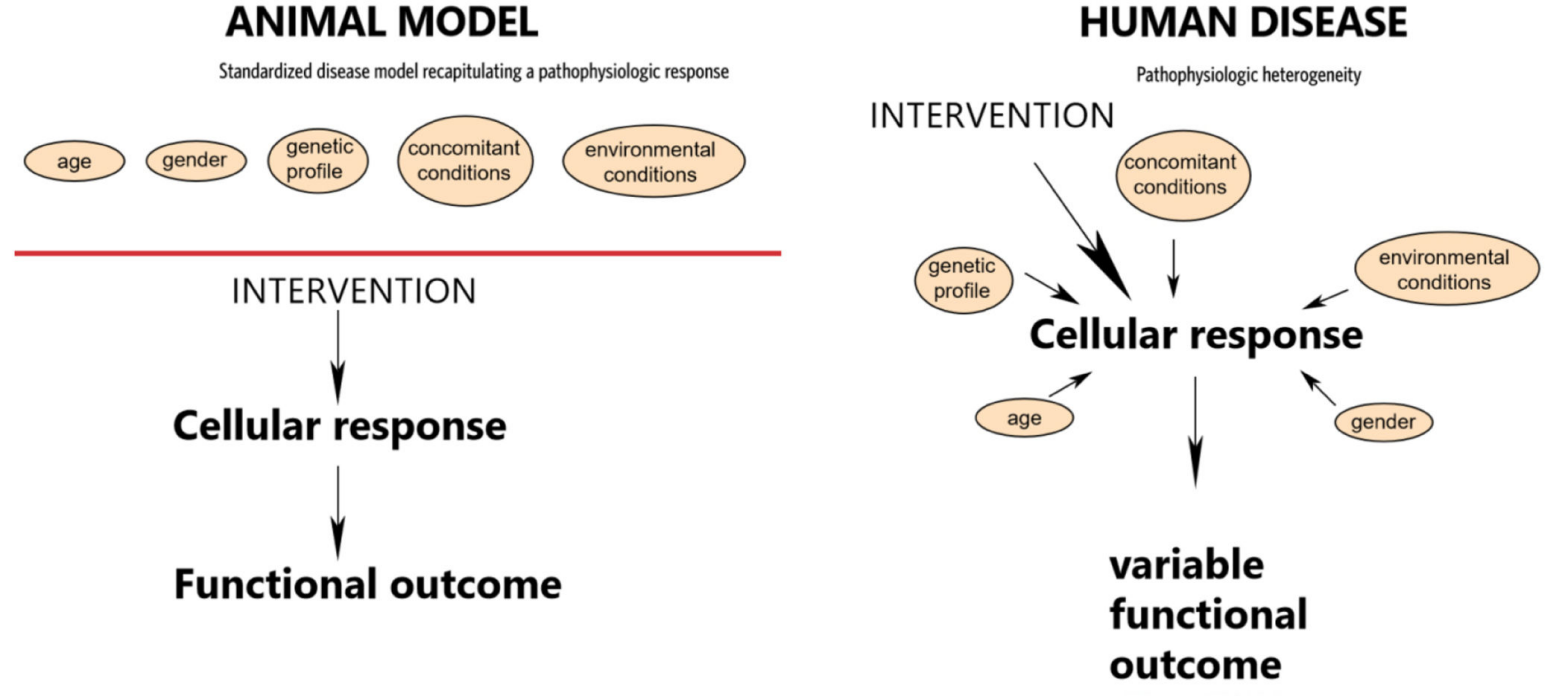
Translational failures are often due to the contrasting characteristics of animal model investigations and of interventional therapeutic studies in human patients. Animal model studies are excellent tools for testing a hypothesis on the role of a cellular mechanism, or of a specific molecular signal in the pathophysiology of disease. To achieve these goals, animal model investigations are designed to minimize variability by using standardized protocols that control the impact of comorbid conditions, genetic differences or environmental conditions. These studies provide valuable information on cell biological mechanisms and have potential implications for organ function, but are of much more limited value in predicting the outcome of a similar intervention in the clinical context. In complex multifactorial human diseases, patient populations exhibit remarkable pathophysiologic heterogeneity. Moreover, differences in age, gender, genetic substrate, the presence or absence of concomitant diseases, treatment with other agents, environmental conditions, may directly affect cellular responses, affecting clinical outcomes. No animal model can recapitulate the pathophysiologic heterogeneity of human disease. Thus, animal model investigations should optimally be used for cell biological dissection, and not for the prediction of therapeutic outcomes. Moreover, in the clinical context, stratification of patients with complex clinical syndromes (such as heart failure, or chronic renal insufficiency) to pathophysiologically distinct subpopulations with well-defined molecular perturbations may improve the chances for successful translation.

Third, animal studies cannot recapitulate the many different comorbid diseases, environmental conditions and genetic predispositions that contribute to the variability of responses in human populations. Due to these limitations, the major strength of a well-designed animal study is the ability to dissect a mechanism, or to contribute to the cell biological paradigm through the use of loss- or gain-of-function interventions. A fundamental aspect of this hypothesis-testing approach is the minimization of variability. In a well-designed animal study aimed at testing a specific hypothesis on the role of a molecular signal or interaction, animals are typically age- and gender-matched and have identical genetic profiles. All other aspects of variability that could have an impact on the cell biological response are eliminated in order to study the role of a very specific molecular interaction. The philosophy of this mechanism-dissecting approach comes in sharp contrast with the marked variability observed in the clinical context [Figure 2]. Human patients exhibit marked differences in genetic substrate, the pattern and characteristics of the disease, and the presence or absence of concomitant conditions. This remarkable heterogeneity has profound consequences on the outcome and cannot be recapitulated in an animal model.

## Towards pathophysiologic definitions of human disease

Traditional classifications of diseases are based predominantly on clinical manifestations and functional perturbations, and much less on pathophysiologic insights. Although this approach remains practical and useful, allowing physicians to navigate through diagnostic and therapeutic algorithms, it poses significant limitations in the development of novel therapeutics. Most common diseases exhibit remarkable pathophysiological heterogeneity. Several distinct cell biological perturbations may result in similar patterns of organ dysfunction, leading to the development of the same clinical syndrome. Our growing understanding of the cellular and molecular mechanisms of diseases, and technical advances in molecular imaging and proteomic strategies for the identification of novel biomarkers may allow pathophysiologic stratification of patients with functionally-defined conditions. Identification of patient subpopulations on the basis of the underlying pathophysiologic mechanisms would represent a major advance in the development of rational mechanism-driven therapeutics.
